# Expression and Rhythmic Modulation of Circulating MicroRNAs Targeting the Clock Gene *Bmal1* in Mice

**DOI:** 10.1371/journal.pone.0022586

**Published:** 2011-07-22

**Authors:** Vikram R. Shende, Marianna M. Goldrick, Suchitra Ramani, David J. Earnest

**Affiliations:** 1 Department of Biology, Texas A&M University, College Station, Texas, United States of America; 2 Center for Biological Clocks Research, Texas A&M University, College Station, Texas, United States of America; 3 Department of Neuroscience and Experimental Therapeutics, College of Medicine, Texas A&M University Health Science Center, College Station, Texas, United States of America; 4 BIOO Scientific, Austin, Texas, United States of America; Victor Chang Cardiac Research Institute (VCCRI), Australia

## Abstract

MicroRNAs (miRNAs) interact with 3′ untranslated region (UTR) elements of target genes to regulate mRNA stability or translation and thus play a role in regulating many different biological processes, including circadian rhythms. However, specific miRNAs mediating the regulation of essential clock genes remain largely unknown. Because vesicles containing membrane-bound miRNAs are present in the circulatory system, we examined miRNAs predicted to target the clock gene, *Bmal1*, for evidence of rhythmic fluctuations in circulating levels and modulatory effects on the 3′ UTR activity of *Bmal1*. A number of miRNAs with *Bmal1* as a predicted target were expressed in the serum of mice exposed to LD 12∶12 and of these miRNAs, miR-152 and miR-494 but not miR-142-3p were marked by diurnal oscillations with bimodal peaks in expression occurring near the middle of the day and 8 or 12 hr later during the night. Co-transfection of pre-miR over-expression constructs for miR-494 and miR-142-3p in HEK293 cells had significant effects in repressing luciferase-reported *Bmal1* 3′ UTR activity by as much as 60%, suggesting that these miRNAs may function as post-transcriptional modulators of *Bmal1*. In conjunction with previous studies implicating miRNAs as extracellular regulatory signals, our results suggest that circulating miRNAs may play a role in the regulation of the molecular clockworks in peripheral circadian oscillators.

## Introduction

MicroRNAs are small non-coding RNAs that have been identified in many different organisms ranging from *Drosophila* to humans [1] and implicated in the regulation of a wide array of biological processes. Mature miRNAs are small RNA molecules, typically 19–25 nucleotides long, derived from sequential RNase III-dependent cleavages of longer transcripts [2], [3]. In the cytoplasm, mature miRNAs associate with components of the RNA-induced Silencing Complex (RISC) and interact with miRNA-recognition elements (MRE's) in the 3′ UTRs of target mRNAs. Mismatches or gaps in the base-pairing interactions between the miRNA-mRNA duplex result in translational repression and/or mRNA de-stabilization [4], [5]. In humans, it has been estimated that the number of unique miRNAs exceeds 1000 [6] and that 20–30% of the transcriptome is subject to miRNA-targeted regulation [7], [8]. Although miRNAs target and regulate specific mRNA transcripts via intracellular mechanisms, recent evidence for their presence in vesicles circulating in the blood of humans [9] raises the possibility that miRNAs may also function as extracellular or secreted regulatory signals that mediate communication between cells [10]. In accord with their role in modulating the transcriptome and proteome, miRNAs play an integral role in important biological processes like development, metabolism and cancer biology.

Recent studies have also implicated miRNAs in the regulation of the circadian timekeeping mechanism in the mammalian suprachiasmatic nuclei (SCN). Brain, muscle ARNT-like protein 1 (*Bmal1*), circadian locomotor output cycles kaput (*Clock*), as well as the Period (*Per1* and *Per2*) and Cryptochrome (*Cry*) genes comprise the “gears” of the molecular clockworks common to both the SCN and peripheral tissues [11], [12], [13]. Interactions between these clock components form positive- and negative-feedback loops in which gene transcription is rhythmically regulated by their protein products, with exception of *Clock*. For example, rhythmic increases in *Bmal1* transcription and the formation of CLOCK∶BMAL1 heterodimers positively regulate the rhythmic transcription of the *Per* and *Cry* genes [14]. In turn, the increases in PER and CRY proteins lead to the formation of heterodimers that interact with the CLOCK∶BMAL1 complex and negatively feedback on their own transcription. CLOCK∶BMAL1 complexes also mediate the regulation of clock-controlled outputs that provide for the rhythmic programming of downstream processes. MiRNA function in SCN-mediated regulation of circadian rhythms is supported by observations indicating that miR-219 and miR-132 are rhythmically expressed in the SCN and that antagonism of these miRNAs within the SCN region respectively increases the circadian period of behavioral rhythmicity and attenuates circadian photoentrainment [15]. Other applications of mouse, *Drosophila* and chicken models provide further evidence for the role of miRNAs in the regulation of circadian rhythms in gene expression or behavior [16], [17], [18]. However, evidence for the role of specific miRNAs as bona fide modulators of core clock genes is limited.

Because rhythmicity is a prevalent property among core and regulatory elements of the circadian clockworks in most cells and tissues throughout the body, we explored the possible timekeeping function of miRNAs in the periphery by initially determining whether specific miRNAs with clock genes as predicted targets are expressed in serum and whether their expression is marked by daily fluctuations. Our analysis focused on miRNAs that are predicted to target the 3′ UTR of *Bmal1* mRNA because this clock gene is unique among core clock elements as knockout of *Bmal1* alone produces complete loss of circadian rhythmicity [19]. Specifically, experiments were conducted to explore miRNA function in the circadian clockworks by: 1) first identifying miRNAs predicted to target core and ancillary clock genes; 2) determining whether expression of specific miRNAs that target *Bmal1* oscillate in mouse serum *in vivo*; and 3) examining the effects of candidate miRNA over-expression on *Bmal1* 3′ UTR activity.

## Materials and Methods

### Experiment 1: Temporal Profiling of miRNA levels in mouse serum

#### Animals

Experimental subjects were 20 male Balb/C mice at 8–10 weeks of age. All animals were born and reared in the animal facility at BIOO Scientific under a standard 12 h light∶12 h dark photoperiod (LD 12∶12; lights-on at 0700 hr). Prior to experimental analysis, animals were housed 2–3 per cage. Access to food and water was provided *ad libitum* and periodic animal care was performed at random times.

#### Ethics Statement

All animal procedures used in this study were conducted in compliance with protocol B002 as approved by the Institutional Animal Care and Use Committee at BIOO Scientific Corp.

#### Blood collection and fractionation

To determine whether miRNAs are expressed and fluctuate rhythmically in the serum, blood samples were collected at 4 hour intervals from mice maintained in a LD 12∶12 cycle. At each timepoint, blood was collected by cardiac puncture from 3–4 mice that were anesthetized with 2,2,2-tribromoethanol (250 mg/kg, intraperitoneal; Sigma) and sacrificed by cervical dislocation. Sampling procedures during the dark phase of the LD 12∶12 cycle (Zeitgeber Time [ZT] 12–24) were conducted under dim red light (Kodak filter GBX-2). Blood was allowed to clot at room temperature for 10–15 minutes. Adhesions between the clot and collection tube were gently detached by “rimming the clot” to minimize hemolysis and then samples were centrifuged for 10 minutes in a swinging bucket microcentrifuge (Eppendorf) at 3,000× g to separate cellular and non-cellular fractions. Immediately following centrifugation, the serum layer was carefully aspirated off, mixed with three equivalent volumes of TRI reagent (Ambion) and stored at −20°C until further processing.

To fractionate the white blood cells (WBCs), the clot was disaggregated in phosphate buffered saline (PBS) and the fluid layer containing suspended WBCs and red blood cells (RBCs) was collected. Following centrifugation for 30 seconds at 3,000× g, the cell pellet was resuspended in distilled water for ∼10 sec to lyse RBCs by osmotic shock, and then mixed with 10× PBS to restore physiological ionic strength and prevent WBC lysis. The WBC pellet was recovered by centrifugation at 3,000× g for 40 sec, lysed in 1 ml BiooPure RNA Isolation Reagent (BIOO Scientific, Austin, TX) and stored at −20°C until further processing.

#### RNA extractions

Total RNA was subsequently extracted from the serum and WBC lysates according to manufacturer's protocols, with the exception that 50 ug of linear acrylamide was added as a co-precipitant to the aqueous phase before addition of isopropanol. This modification enhances recovery of small amounts of nucleic acids [20]. RNA samples were suspended in 50 ul 0.1 mM EDTA and dissolved by heating for 5 min at 65°C. Total RNA was estimated using Nanodrop ND2000 (Thermo Scientific).

#### Real-time PCR

Quantitative real-time PCR analysis was conducted using Taqman microRNA assays (Applied Biosystems). RNA from individual samples was first reverse transcribed using target-specific stem-loop primers and Taqman MicroRNA Reverse Transcription Kit. All assays were performed according to manufacturer's protocols, using 20 ng of total RNA as input with the exception that 90 ng input RNA was used for reverse transcription of miR-494. For analysis of miRNA expression, the cDNA equivalent of 2 ng of total RNA was PCR amplified in an ABI PRISM 7500 Fast sequence detection system using the following standard conditions: 1) heating at 95°C for 10 min, and 2) amplification over 40 cycles at 95°C for 15 sec and 60°C for 1 min. This analysis was conducted concurrently on duplicate aliquots of RT product from each sample. miR-16 was also amplified from the same samples using identical parameters to control for differences in sample RNA content and reverse-transcription efficiencies because: 1) this miRNA has provided a good standard for normalization and comparisons of relative abundance in previous studies [21], [22]; and 2) ANOVA analysis indicates that miR-16 levels in the serum exhibit no significant variation (*p* = 0.19) over the 24-hour time course for sampling (data not shown). Using the comparative C_T_ method described in the ABI Prism 7700 Sequence Detection System User Bulletin #2 (PE-ABI), the relative abundance for a given miRNA was calculated by normalization first to corresponding miR-16 levels in each sample and then to a calibrator consisting of pooled cDNA from multiple samples over the entire time series. All TaqMan miRNA assays used in this study exhibited PCR efficiencies of 95–101.6% (Figure S1).

Relative quantification of 18s rRNA abundance was performed on some serum and WBC samples using SYBR-Green real-time PCR technology (ABI) as described previously [23], [24]. To generate single-strand cDNAs, total RNA (250 ng) from individual samples was reverse transcribed using random hexamers and Superscript III reverse transcriptase (Invitrogen). 18s rRNA was PCR amplified using the cDNA equivalent of 2.5 ng of total RNA. PCR analysis was performed on duplicate aliquots of each sample using the ABI PRISM 7500 Fast sequence detection system and the following conditions: 1) serial heating at 50°C for 2 min and 95°C for 10 min, 2) amplification over 40 cycles at 95°C for 15 sec and 60°C for 1 min, and 3) dissociation at 95°C for 15 sec, 60°C for 1 min, 95°C for 15 sec and 60°C for 15 sec. Relative differences in 18s rRNA abundance were established by comparing serum and WBC determinations to a standard curve that was generated using pooled cDNA from all samples. The following primers were used for the real-time PCR analysis: m18s rRNA forward: 5′-ATGGCCGTTCTTAGTTGGTG -3′; m18s rRNA reverse: 5′-CGCTGAGCCAGTCAGTGTAG -3′.

To estimate the number of copies of representative miRNAs in serum samples, synthetic single-stranded RNA oligonucleotides encoding the mature miRNA sequences for miR-16 and miR-152 were purchased from Integrated DNA Technologies, Inc. Mature miRNA sequence information was procured from miRBASE (release 16.0; Sept. 2010). These synthetic miRNAs were used in a dilution series ranging from 1 molecule/µl to 10^10^ molecules/µl to generate standard curves for quantification of molecules of miR-16 and miR-152. Standard curves derived from concentrations yielding Ct values within the linear range were used to estimate the number of copies of miR-16 and miR-152 in the input RNA from ZT7 serum samples that were simultaneously reverse-transcribed, PCR-amplified and analyzed on the same plate. Absolute copy number of miRNAs per microliter of serum was extrapolated using known information on the amount of input RNA (10 ng) and total extracted RNA (1500–4500 ng) relative to the specific volume collected (200–300 µl) for each serum sample.

### Experiment 2: MiRNA regulation of *Bmal1* 3′ UTR activity

#### *mBmal1* 3′ UTR luciferase reporter construct

miTarget™ miRNA Target Sequence 3′ UTR Expression Clone for *Bmal1* was purchased from Genecopoeia . This expression clone contains *Bmal1* (Accession: NM_007489.3) 3′ UTR sequence inserted in the pEZX-MT01 vector downstream of a firefly luciferase gene under the control of an SV40 enhancer generating a chimeric transcript that consists of the luciferase coding and *Bmal1* 3′ UTR sequences (Figure S2). The pEZX-MT01 vector also contains the *Renilla* luciferase gene under the control of a CMV promoter to provide for dual analysis of firefly and *Renilla* luciferase activities in individual samples and to normalize firefly luciferase signal intensities and account for potential differences in transfection efficiencies across control and experimental cultures. To determine the specificity of miRNA interactions with the *Bmal1* 3′ UTR, similar analyses were performed using miRNA 3′ UTR target control vector (Genecopoeia; CmiT000001-MT01), which consists of the pEZX-MT01 vector without a 3′ UTR tagged to the firefly coding sequence. miTarget™ miRNA Target Sequence 3′ UTR Expression Clone encoding the *cKit* 3′ UTR (Accession: NM_021099.2; generously provided by Dr. Rajesh Miranda, Texas A&M University Health Science Center) was also used as an additional control. Based on the results from Targetscan analysis, the *cKit* 3′ UTR is predicted to contain a target site for miR-494, but not for miR-142-3p or miR-152. The *cKit* 3′ UTR also contains a predicted target site for miR-142-5p, the antisense transcript of miR-142-3p.

Transformed *E. coli* cells (Genecopoeia) were grown on kanamycin (final conc. = 50 ug/ml) containing imMedia agar plates (Invitrogen). A single isolated colony was propagated in imMedia Kan^+^ (final conc. = 50 ug/ml) liquid medium and plasmid was extracted using EndoFree Plasmid Maxi kit (Qiagen). The extracted pEZX-MT01 *Bmal1* 3′ UTR expression plasmid was sequenced to verify expression and accuracy of the *Bmal1* 3′ UTR sequence using the following primers: Forward: 5′-GATCCGCGAGATCCTGAT-3′; Reverse: 5′-TTGGCGTTACTATGGGAACAT-3′. Similar procedures were followed for isolation of the pEZX-MT01 control vector and the pEZX-MT01 *cKit* 3′ UTR expression vector.

#### Cell culture and transfections

Human embryonic kidney cells (HEK293) at passage 12–15 were used for experimental analysis of miRNA regulation of *Bmal1* 3′ UTR activity. Cells were seeded on 60-mm cell culture dishes (Corning) and maintained at 37°C and 5% CO_2_ in Dulbecco's minimum essential medium (DMEM; Invitrogen) without antibiotics and supplemented with 10% Fetal Bovine Serum (FBS; HyClone, Logan, UT) and 292 ug/mL L-glutamine. Medium was changed at 48-hour intervals and confluent cultures were split 1∶4 or 1∶5 every 3–4 days. Prior to experimentation, cells were seeded onto 24-well plates in DMEM supplemented with 5% FBS. 24 hours later, co-transfection of verified plasmid DNA clones (0.4 ug) with either individual pre-miRs, or paired combinations of pre-miR constructs for miR-494, miR-152, or miR-142-3p (final conc. 33 nM/well; ABI) was performed using Lipofectamine 2000 (Invitrogen) according to the manufacturer's protocols. To compare basal luciferase reporter activity across different pEZX-MT01 vectors and evaluate the potential influence of endogenous miRNAs, parallel analysis was performed on HEK293 cells in which either the pEZX-MT01 control vector or the *Bmal1* 3′ UTR vector was co-transfected with a non-targeting control miRNA. Following incubation with transfection reagents for 5 hours, the medium was replaced and 48 hours later, lysates of HEK293 cultures from all treatment groups (n = 4) were collected using Passive Lysis Buffer (Promega). Lysate samples were stored at −20°C and later firefly luciferase activity was analyzed relative to *Renilla* luciferase activity in the same sample using dual-luciferase reporter assay system (Promega). Luminescence was measured in counts per second using a LumiCount microplate luminometer (AL10000; Packard Bioscience).

#### Statistical analyses

The temporal patterns of miRNA expression in serum were examined for evidence of circadian variation using statistical analyses that have been used previously for this purpose [23], [25], [26]. Time-dependent fluctuations in miR-494, miR-152 and miR-142-3p expression were first identified by one-way analysis of variance (ANOVA). Paired comparisons between peak values and those observed during the preceding or succeeding minimum were analyzed *post hoc* for statistical differences using the Newman-Keuls sequential range test. The α-value was set at 0.05 for these *post hoc* analyses.

Independent *t*-tests were performed on serum and WBC 18s and miRNA comparisons, and on normalized luminescence data to determine the significance of pre-miR and control-miR treatment on luciferase-reported *Bmal1* 3′ UTR activity. The α-value was set at 0.01 for independent *t*-tests.

## Results

### Experiment 1: Temporal Profiling of miRNA levels in mouse serum

**Figure 1:**
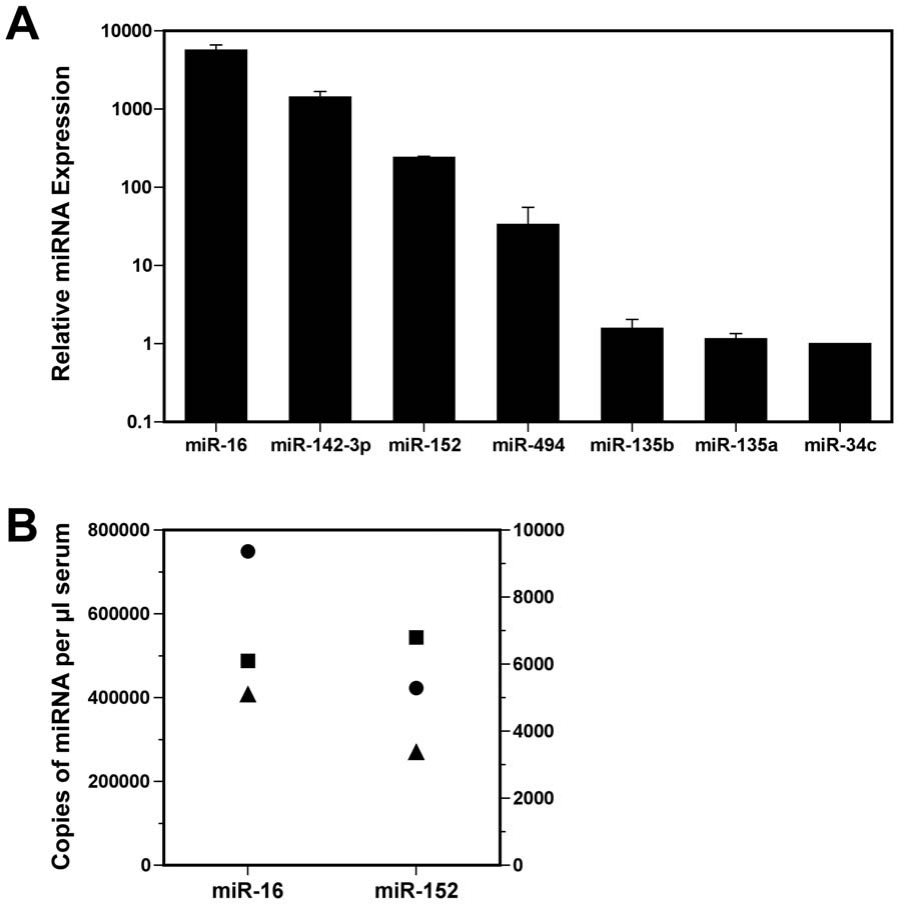
Comparison of circulating miRNAs predicted to target *Bmal1* or other genes regulating *Bmal1* expression with a highly abundant miRNA in serum, miR-16. (**A**) Relative expression of miR-16, miR-142-3p, miR-152, miR-494, miR-135b, miR-135a and miR-34c in serum samples collected from mice at ZT 7 (n = 3). Bars denote real-time PCR determinations of serum miRNA levels (mean ± SEM) and the values are plotted using a logarithmic scale in comparison with the average for miR-34c expression. (**B**) Quantification of miR-16 and miR-152 expression in serum collected from mice at ZT 7 (n = 3). Symbols denote determinations (in duplicate) of the number of copies/µl serum in each sample that were extrapolated by comparing the Ct values for experimental samples with standard curves consisting of a dilution series of known quantities of synthetic miR-16 and miR-152 analyzed on the same plate.

**Figure 2:**
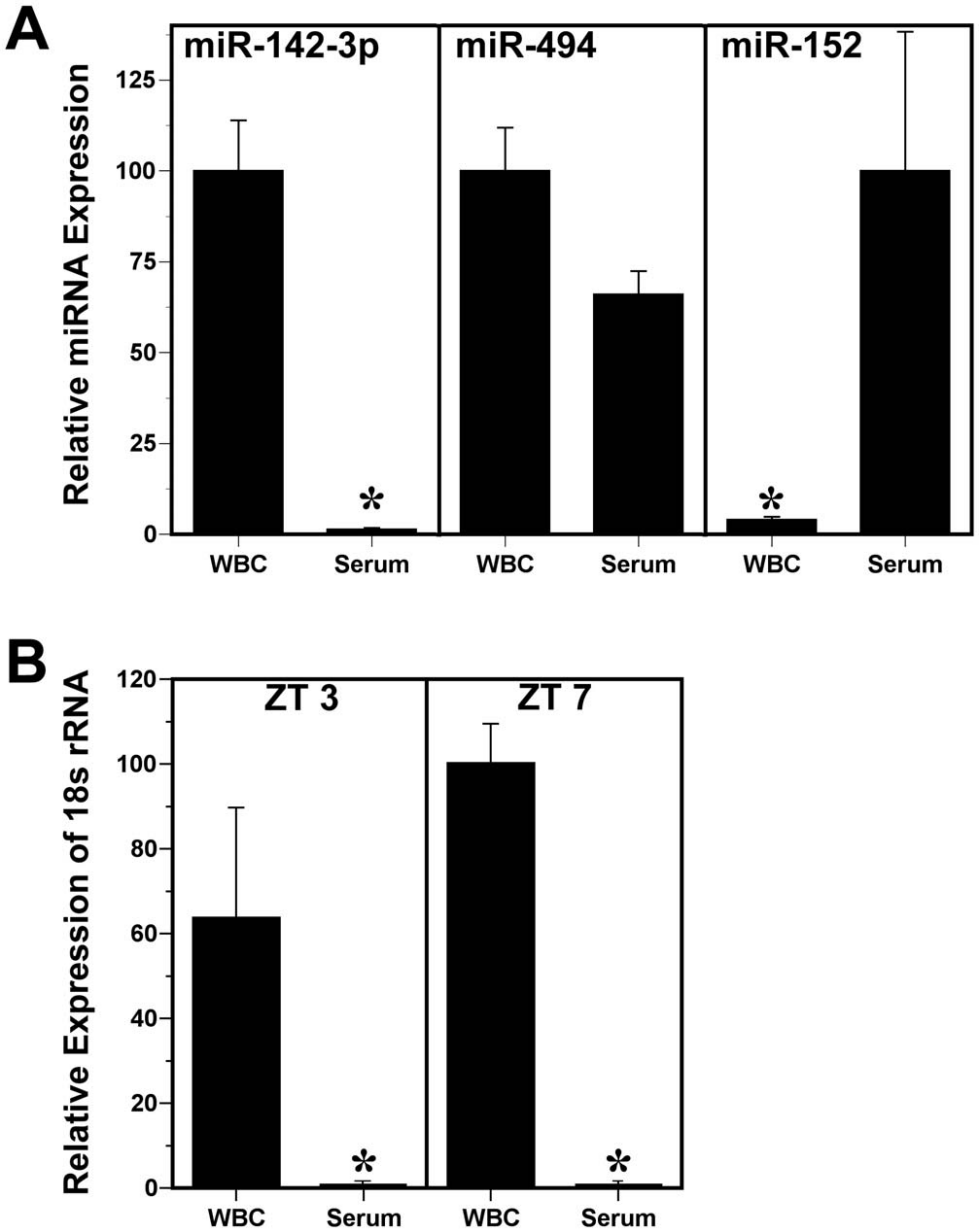
Comparison of miRNA and 18s rRNA levels in the serum and corresponding white blood cell (WBC) fractions. (**A**) Bars denote real-time PCR determinations of serum and WBC miRNA levels (mean ± SEM) in blood samples collected from mice (n = 3) at ZT 7. The plotted values correspond to the ratios of fraction-specific miR-142-3p, miR-494 and miR-152 signal and are represented as a percentage of the maximal value obtained among the serum and WBC fractions. Asterisks denote comparisons in which the relative expression of miRNA signal in the WBC fraction was significantly different (*p*<0.05) from that observed in serum samples. (**B**) Bars denote real-time PCR determinations of 18s rRNA levels (mean ± SEM) in serum and WBC fractions of blood samples collected from mice (n = 3–4) at ZT 3 and ZT 7. The plotted values are represented as a percentage of the average for the WBC fraction at ZT 7. Asterisks denote time-specific comparisons (ZT 3 and ZT 7) of 18s rRNA signal in which relative expression in the WBC group was significantly greater (*p*<0.01) than that observed in serum samples.

Three target prediction databases (microcosm [27], [28], Targetscan [7] and MiRanda [29]) were used to identify potential miRNAs targeting mammalian clock genes. Because *Bmal1* is the only clock gene in which null mutation produces arrhythmicity [19], we focused on a subset of miRNAs that expresses consensus recognition sequences for the 3′ UTR of either *Bmal1* or for other genes that regulate *Bmal1* expression in the molecular feedback loops comprising the circadian clockworks (Table 1). The *Bmal1* 3′ UTR was predicted to contain miR-152, miR-142-3p and miR-494 target sites that are located at nucleotide positions 88–108, 335–357 and 473–495, respectively (Figure S3). It is interesting that in addition to their putative targeting of the *Bmal1* 3′ UTR, miR-152 and miR-494 were also predicted to interact with its primary partner *Clock* or with transcriptional activators, retinoic acid-related orphan receptors alpha and beta (*Rorα*, *Rorβ*). We next determined whether any of the identified miRNAs predicted to target *Bmal1* or other components of the clock feedback loops were expressed in serum. All of the candidate miRNAs were detected in serum during the daytime of LD 12∶12 cycle (ZT 7), but their circulating levels spanned a wide range. miR-142-3p, miR-152, miR-494, miR-135b, miR-135a and miR-34c were found in descending order of abundance in the serum (Fig. 1A). Consistent with previous observations on its circulating levels in humans [9], miR-16 was highly abundant relative to other miRNAs detected in mouse serum. Quantitative analysis of miR-16 and miR-152 levels revealed that the estimated concentrations of these miRNAs were 408,000–749,000 and 3,400–6,800 copies/µl serum (Fig. 1B), respectively. Using this information as a relative index of abundance, miR-142-3p, miR-152 and miR-494 appear to represent species of moderate to low expression in serum. To gauge the relationship between serum and cellular miRNA levels, we also analyzed the WBC fractions of individual blood samples for expression of specific miRNAs predicted to target *Bmal1*. These components of blood exhibited variable differences in miRNA levels such that miR-142-3p and miR-494 expression were lower (77-fold and 1.5-fold, respectively) and miR-152 was higher (25-fold) in serum than in WBCs (Fig. 2A). The differential expression of miR-142-3p in WBCs is not surprising because this miRNA is highly abundant in hematopoietic cell lineages [30].

**Table 1:** Application of target prediction programs to identify candidate miRNAs expected to target *Bmal1* or other genes that regulate *Bmal1* expression.

	mmu-miR-142-3p	mmu-miR-152	mmu-miR-135b	mmu-miR-135a	mmu-miR-34c	mmu-miR-494
**MicroCosm**	*Bmal1*	*Bmal1, Rorβ*	-	-	*Reverbα*	*Bmal1, Rorβ*
**TargetScan**	*Bmal1*	-	-	-	-	*Bmal1, Clock*
**MiRanda**	*Bmal1*	*Bmal1, Rorα, Rorβ*	*Rorα, Rorβ*	*Rorα, Rorβ*	*Per2*	*Bmal1, Per2, Rorβ*

Because recent findings indicate that despite their abundant expression in the cytoplasm, 18s and 28s rRNA are absent in RNA extracted from circulating exosomes [31], 18s rRNA levels were analyzed in serum and WBC fractions of blood samples collected from mice (n = 3–4) at ZT3 and ZT7 to confirm that the detected small RNAs reflect serum expression, rather than artifact associated with cellular lysis during sample preparation. Consistent with the observations of Valadi *et al.* [31], serum levels of 18s rRNA were negligible and at the limits of detection with real-time PCR analysis. Relative expression levels of 18s rRNA in the serum were 26,000–79,000-fold lower than those found in WBC fractions (Fig. 2B), indicating that the observed miRNA signals in serum are derived from extracellular or vesicle-encapsulated RNA, and not from lysed or intact leukocytes or other cells, in the blood samples.

**Figure 3:**
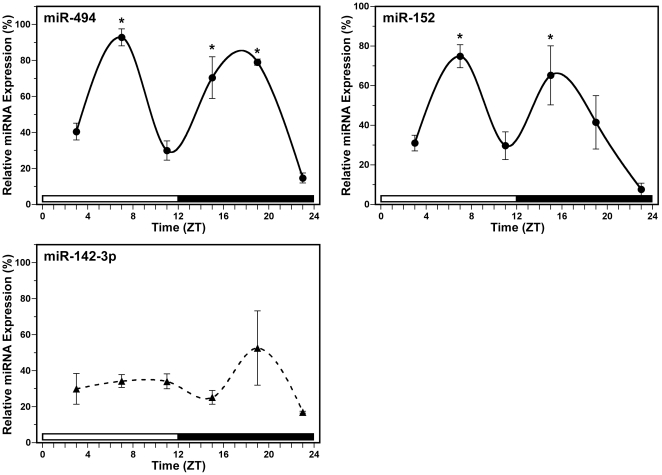
Temporal patterns of miR-494, miR-152 and miR-142-3p expression in mouse serum. Symbols denote real-time PCR determinations of miRNA levels (mean ± SEM) in serum collected at 4-hour intervals from mice (n = 3–4) during exposure to a LD 12∶12 cycle. The plotted values correspond to the ratios of miR-494 (top left), miR-152 (top right) and miR-142-3p (bottom) signal normalized to miR-16 levels in each sample and are represented as a percentage of the maximal value obtained for each miRNA. Asterisks indicate time points during which peak values for serum expression of a given miRNA were significantly greater (*p*<0.05) than those observed during preceding and succeeding minima.

Daily profiles of miRNA expression in serum were assessed to determine whether circulating levels of mature miRNAs that are predicted to target mouse *Bmal1* or other genes regulating this key component of the molecular clockworks, miR-494, miR-152 and miR-142-3p, oscillate rhythmically. In mice exposed to LD 12∶12, diurnal fluctuations were observed in the relative expression of miR-494 and miR-152 (normalized to miR-16) in the serum (Fig. 3). The rhythm in circulating levels of miR-494 was marked by a bimodal pattern in which the first peak in serum expression occurred around mid-day at ZT 7 and was followed by a secondary peak during the night around ZT 19. For the diurnal oscillation in miR-494 expression, the bimodal peaks in serum levels at ZT 7 and ZT 19 were significantly (*p*<0.05) and about 2- to 5-fold greater than those observed during the preceding and succeeding minima. Similar to the temporal profile for miR-494 expression, serum levels of miR-152 were characterized by diurnal variation with bimodal peaks (Fig. 3). The first zenith in miR-152 expression occurred again at ZT 7 and was followed by a secondary peak in mature miRNA levels at ZT 15. The amplitude of rhythmic miR-152 expression in the serum was robust, with 2- to 8-fold differences between peak and trough values. The bimodal peaks in circulating levels of miR-152 at ZT7 and ZT15 were significantly greater (*p*<0.05) than the preceding and succeeding minima. In contrast to other tested miRNAs, miR-142-3p levels in the serum exhibited no evidence of diurnal fluctuations (Fig. 3). In comparison with miR-494 and miR-152, serum levels of miR-142-3p were relatively high with no significant variation over time (*p* = 0.377).

### Experiment 2: MiRNA regulation of *Bmal1* 3′ UTR activity

**Figure 4:**
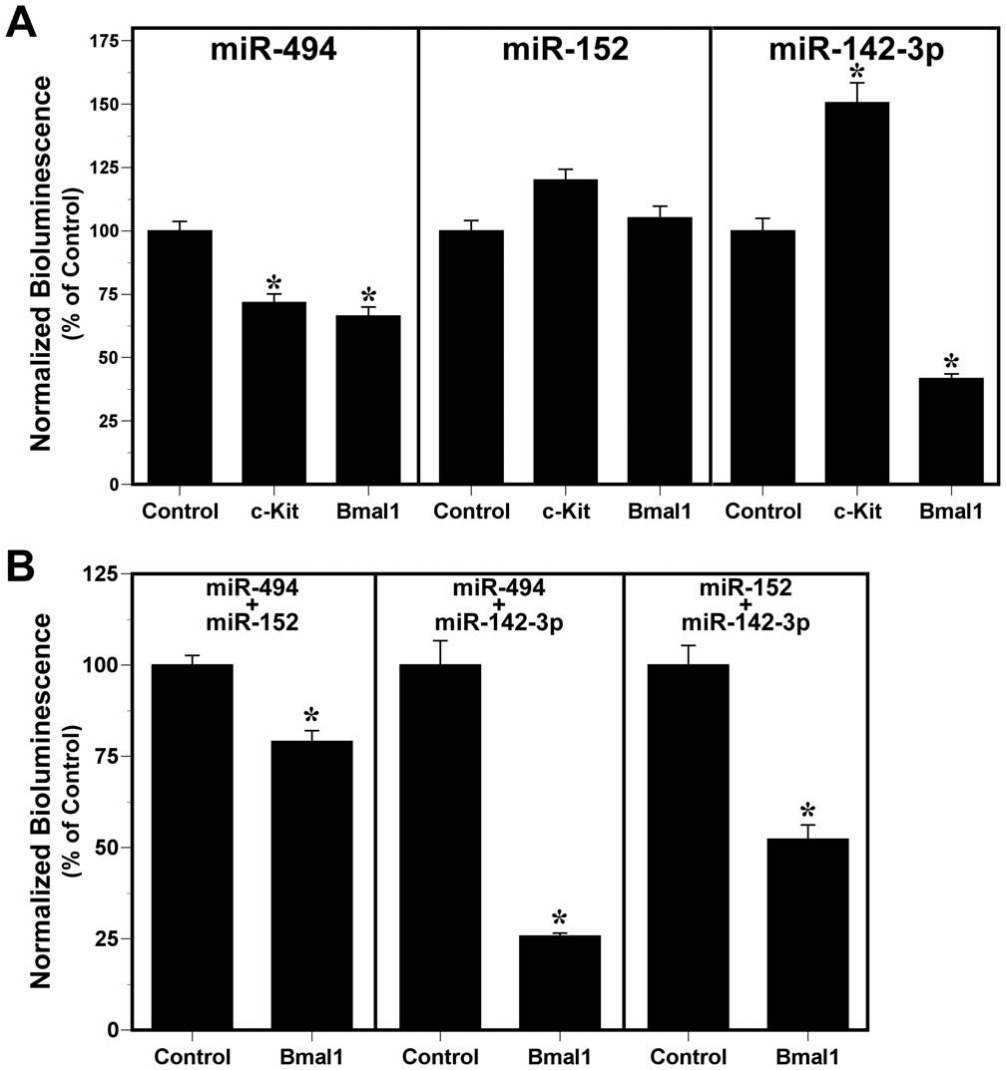
Independent and combinatorial effects of miR-494, miR-152 and miR-142-3p over-expression on *Bmal1* 3′ UTR activity. Bars denote mean (±SEM) determinations of luciferase bioluminescence for each treatment group (n = 4). The plotted values correspond to the ratios of firefly luciferase signal normalized to *Renilla* luciferase activity in the same sample and are represented as a percentage of the average signal for control vector transfectants. (**A**) Normalized bioluminescence from HEK293 cells expressing the pEZX-MT01 control vector, or pEZX-MT01 vector containing either *cKit* 3′ UTR or *Bmal1* 3′ UTR in response to treatment with pre-miR constructs (33 nM) for miR-494, miR-152 and miR-142-3p. (**B**) Normalized bioluminescence from HEK293 cells expressing the pEZX-MT01 control vector or pEZX-MT01 vector containing the *Bmal1* 3′ UTR in response to treatment with paired combinations of these pre-miR constructs (33 nM; miR-494+miR-152, miR-494+miR-142-3p, miR-152+miR-142-3p).

Since the observed circadian fluctuations in serum levels of miRNAs predicted to target *Bmal1* is suggestive of their involvement in circadian timekeeping mechanisms, we used an *in vitro* reporter assay to examine the effects of miR-494, miR-152 and even miR-142-3p on *Bmal1* expression via targeting of the 3′ UTR of this clock gene. Bioluminescence was analyzed in HEK293 cells co-transfected with the pEZX-MT01 *Bmal1* 3′ UTR expression vector and pre-miR constructs for miR-494, miR-152, or miR-142-3p (n = 4). To control for non-specific interactions between pre-miRs and the luciferase reporter in the pEZX-MT01 vector, parallel analysis was performed on HEK293 cells co-transfected with these pre-miR constructs and the pEZX-MT01 control vector which does not contain a 3′ UTR tagged to the firefly coding sequence (n = 4). In HEK293 cells co-transfected with *Bmal1* 3′ UTR expression vector, over-expression of miR-494 or miR-142-3p, but not miR-152, produced significant decreases (*p*<0.01) in luciferase-mediated bioluminescence relative to that found in cells transfected with the control vector (Fig. 4A). Treatment with pre-miR constructs for miR-494 or miR-142-3p repressed *Bmal1* 3′ UTR-mediated bioluminescence by about 35% and 60%, respectively, in comparison with control transfections. Transfection with a non-targeting miRNA had no significant effect on luciferase-mediated bioluminescence in *Bmal1* 3′ UTR-expressing cells relative to that found in cells transfected with the control vector, suggesting that basal reporter activity is similar between the *Bmal1* 3′ UTR and control vectors and that the observed repression of the *Bmal1* 3′ UTR is specific for miR-494 and miR-142-3p.

Pre-miR interactions with the 3′ UTR of a non-clock gene containing predicted target sites for miR-494 but not miR-152 or miR-142-3p were explored by analyzing bioluminescence from HEK293 cells co-transfected with the pEZX-MT01 vector expressing the *cKit* 3′ UTR and pre-miR constructs for these miRNAs (n = 4). Consistent with target prediction data indicating that the *cKit* 3′ UTR contains a predicted target site for miR-494, overexpression of miR-494 in pEZX-MT01 *cKit* 3′ UTR-transfected cells yielded a significant reduction (*p*<0.01) in luciferase-reported bioluminescence relative to that observed in cells co-transfected with this pre-miR and the control vector (Fig. 4A). In response to treatment with pre-miR constructs for miR-494, *cKit* 3′ UTR-mediated bioluminescence was repressed by about 30% in comparison with luciferase reporter expression in cells co-transfected with the control vector. Importantly, treatment with pre-miR constructs for miR-152 had no significant effect in repressing luciferase-mediated bioluminescence in *cKit* 3′ UTR-expressing cells. In fact, overexpression of miR-152 in pEZX-MT01 *cKit* 3′ UTR-transfected cells produced a small increase in bioluminescence relative to that found in control vector-transfected cells. Interestingly, treatment with pre-miR constructs for miR-142-3p caused a significant increase in *cKit* 3′ UTR-mediated bioluminescence relative to the luciferase activity of the control vector. The basis for this inductive effect of miR-142-3p on *cKit* 3′ UTR activity is unknown, but the *cKit* 3′ UTR is predicted to contain a target site for miR-142-5p and overexpression of its antisense transcript, miR-142-3p, may effectively reverse any basal repression derived from interactions of endogenous miR-142-5p with the *cKit* 3′ UTR.

Because previous studies indicate that two different miRNAs can act in concert to simultaneously repress translation of a single mRNA [32], we next examined the combinatorial effects of miR-494, miR-152 and miR-142-3p overexpression in repressing *Bmal1* 3′ UTR activity. For all of the tested miRNA combinations (miR-494+miR-152, miR-494+miR-142-3p, miR-152+miR-142-3p), luciferase-reported bioluminescence was significantly reduced (*p*<0.01) in *Bmal1* 3′ UTR-expressing cells relative to that control vector transfectants (Fig. 4B). Combinatorial effects in repressing luciferase-reported *Bmal1* 3′ UTR activity were lowest in response to miR-494 and miR-152 overexpression (∼21%) and were highest in cells treated with pre-miR constructs for miR-494 and miR-142-3p (∼75%). In conjunction with the observed differences in the repressive effects of individual miRNAs, these results suggest that among the tested transcripts, miR-142-3p is the most potent repressor of *Bmal1* 3′UTR-mediated activity. Moreover, the potentiated repression observed in response to combined treatment with pre-miR constructs for miR-142-3p and miR-494 is compatible with previous evidence for the synergistic regulation of the 3′ UTR for a single gene by two or more miRNA species [32]. Based on the independent and combinatorial effects of miR-494 on *Bmal1* 3′ UTR activity, our results support the possibility that oscillations in serum levels of this miRNA may contribute to local rhythms in the post-transcriptional repression of endogenous *Bmal1* in the periphery.

## Discussion

Circulating levels of many humoral factors, including insulin, adrenalin, leptin, ghrelin and corticosterol, are marked by robust circadian rhythmicity [33], [34]. These hormones and neuroendocrine factors are thought to play a role in the hierarchical organization of the mammalian circadian system with regard to the coordination of rhythmicity between peripheral and CNS oscillators [35], [36]. Although there is now increasing evidence for the presence of circulating miRNAs and their potential implications as biomarkers of pathological and physiological states [9], [37], [38], the current study provides primary evidence indicating that levels of some circulating miRNA species are subject to rhythmic regulation as well. Specifically, our findings indicate that several miRNAs predicted to target core clock genes are also expressed in mouse serum, and that circulating levels of miR-494 and miR-152 are distinguished by diurnal oscillations. Similar to other diurnal and circadian rhythms in various processes, including arterial pressure, neurotransmitter receptors, and circulating levels of hormones [39], [40], [41], serum levels of miR-494 and miR-152 oscillate with bimodal patterns that are thought to reflect the independent rhythmicity of two uncoupled oscillators. It is noteworthy that despite the similarity in the phase of their rhythmic profiles with bimodal peaks occurring near mid-day and during the night, miR-494 and miR-152 are transcribed at different loci in the mouse genome; both are transcribed from a single locus, on chromosome 11 for miR-152 and on chromosome 12 for miR-494. Another distinction is that miR-152 is an intronic miRNA, while miR-494 is part of a large cluster.

The mechanism responsible for the rhythmic variations in serum levels of miR-494 and miR-152 is unknown. Importantly, comparative analysis of 18s rRNA levels in serum and corresponding WBC samples suggests that the miRNA expression and rhythmic profiles detected in serum are not contamination or artifact derived from lysed or intact cells in the circulation. The observed rhythms in circulating levels of miR-494 and miR-152 may correspond to temporal variation in the intracellular production, packaging or endocytic trafficking of these miRNAs and/or their uptake by specific target cells. Alternatively, it is possible that the differential stability of some circulating miRNAs over time may contribute to these serum oscillations.

Recent evidence suggests that miRNAs may act as molecular switches regulating the timing of various biological events [42]. Thus, the present implications for miRNAs in circadian timekeeping seem to represent a logical extension of their known functions. At present, the precise role of miRNAs in either the molecular clockworks or the hierarchical organization of mammalian circadian oscillators is unclear. Several reports indicate that miRNAs may modulate some aspects of circadian pacemaker function and output rhythms [15], [16]. Using *in vitro* analysis of luciferase-reported *Bmal1* 3′ UTR activity to examine the effects of miRNA over-expression, miR-494 and miR-142-3p were identified as potential post-transcriptional repressors of *Bmal1*, for the first time implicating specific miRNAs in the regulation of this integral molecular component of the mammalian circadian clock.

The phase relationship between the diurnal rhythms in circulating levels of these miRNAs and *Bmal1* oscillations in the periphery is consistent with the potential function of miR-494 in the post-transcriptional regulation of *Bmal1*. In most peripheral tissues, *Bmal1* accumulation follows a circadian profile in which mRNA levels are high from the middle of the night to early morning and remain low throughout the rest of the cycle [43], [44]. Hepatic BMAL1 protein content oscillates in a similar fashion such that the rhythmic peak occurs late in the subjective night and levels rapidly decline near the middle of the subjective day [45]. Thus, the observed oscillations in circulating levels of miR-494 and miR-152 exhibit an interesting relationship with reported circadian profiles for *Bmal1* in the periphery, with bimodal peaks of miRNA expression encompassing times around the rising and falling phases of the *Bmal1* mRNA as well as protein rhythms. In conjunction with our evidence for miR-494-mediated repression of the *Bmal1* 3′ UTR, the bimodal pattern of these serum oscillations may have some significance for the function of miR-494 and other miRNAs regulating core clock components as local and/or systemic cues that fine-tune the circadian harmonics of intercellular interactions and coordinate rhythmicity between autonomous circadian oscillators in peripheral tissues.

Although both miR-494 and miR-142-3p target the same gene, *Bmal1*, overt rhythmicity in extracellular levels may not be the sole criterion determining their role in the mammalian circadian system. Given the abundance of RNases in serum and evidence for the rapid degradation of naked synthetic miRNAs in plasma [9], [46], it seems likely that the endogenous serum miRNAs observed in our experiments are packaged within various types of protective, membrane-bound particles, such as microvesicles and exosomes. Vesicles released into the circulation *in vivo* presumably arise from a wide variety of cells derived from different lineages and subtypes at different stages of maturation, and are distinguished by diverse functions. Recent studies indicate that these secreted vesicles mediate the intercellular communication of specific RNA signals [31], [47], [48], [49] and that vesicle-transmitted miRNAs are functional in recipient cells [47], [48]. Furthermore, different miRNAs are packaged in vesicles and exosomes expressing integral cell membrane proteins derived from their parental cells of origin, and these cell surface markers appear to specify the capture of these particles by certain tissues or cell types. For example, mature dendritic cell derived exosomes that express intercellular adhesion molecule-1 (ICAM1) are captured largely by lymphocyte function-associated antigen-1 (LFA-1) expressing activated T-cells and CD8^+^ dendritic cells, but not by CD8^−^ dendritic cells [50], [51]. Hence, the distinctive functions of different circulating miRNAs in regulating the molecular clockworks or overt circadian rhythms within or between specific tissues may be determined not only by temporal variation in extracellular levels but also by the parental cells from which the miRNA-containing vesicles originate, and by cell-surface receptors on specific target cells that capture these circulating vesicles.

Because a number of processes are subject to circadian regulation in the cardiovascular system, circulating miRNAs may play an important role in the local and/or systemic coordination of circadian rhythms associated with cardiovascular physiology and pathology. The cardiovascular system is distinctly characterized by circadian regulation of various parameters such as arterial pressure, heart rate, and vascular tone [52], [53]. Furthermore, circadian variation is an important factor in the manifestation of cardiovascular pathology, including myocardial infarction, sudden cardiac death and stroke [52]. The role of identified miRNAs with *Bmal1* as a putative target and rhythmic variations in their circulating levels in cardiovascular physiology and disease is currently unknown. However, miR-494 has been recently implicated in cardiac pathophysiological processes because cardiac-specific over-expression of this miRNA reduces myocardial infarction size in response to ischemia/reperfusion-induced cardiac injury [54]. In addition, the recent finding that deletion of several secretory vesicle proteins disrupts circadian rhythms of blood pressure and heart rate in the mouse [55] may have further implications for the role of extracellular miRNAs in regulating cardiovascular physiology. Nevertheless, our results suggest that extracellular miRNAs may play a role in the regulation of peripheral circadian clocks and that circadian profiling and comparison of different serum miRNAs in various disease models may provide a valuable tool in identifying biomarkers for human cardiovascular pathologies associated with circadian rhythm disturbances.

## Supporting Information

Figure S1 **Estimation of qPCR efficiencies for Taqman miRNA assays.** qPCR efficiencies were calculated using the formula; E = 10^(−1/slope of standard curve)^. Percentage efficiencies were calculated using the formula; %E = (E−1)×100. For miR-16 and miR-152 assays (**A**), ‘E’ was calculated based on the slopes of standard curves generated using synthetic single-stranded RNA oligonucleotides. For miR-142-3p and miR-494 assays (**B**), the slopes of standard curves generated using dilutions of a calibrator created from pooled cDNA samples from various timepoints were used to calculate ‘E’.
(TIF)

Figure S2 **Design of pEZX-MT01** ***Bmal1*** **and** ***cKit*** **3′ UTR luciferase reporter vectors.** pEZX-MT01 dual luciferase reporter plasmid construct encodes a chimeric transcript containing the mouse *Bmal1* or *cKit* 3′ UTR tagged to firefly luciferase coding sequence under control of an SV40 enhancer. In comparison, the miRNA 3′ UTR target control vector lacks a 3′ UTR target downstream of the pEZX-MT01 firefly luciferase coding sequence. All vectors contain a kanamycin resistance cassette for selection of bacterial transformants stably expressing the pEZX-MT01 plasmid and the *Renilla* luciferase coding sequence transcribed under control of CMV promoter to normalize firefly luciferase signal intensities across samples.
(TIF)

Figure S3 **Predicted interactions between miR-494, miR-152 and miR-142-3p and the** ***Bmal1*** **3′ UTR.** Diagrammatic representation of predicted interactions between mature (**A**) miR-494, (**B**) miR-152 and (**C**) miR-142-3p with complementary regions of the wild-type *Bmal1* 3′ UTR. Dotted lines indicate potential stabilizing interactions between guanine and thymine bases.
(TIF)
