# Supplementary figures and images for: Expression and Rhythmic Modulation of Circulating MicroRNAs Targeting the Clock Gene Bmal1 in Mice
Source: PLoS One. 2011 Jul 22;6(7):e22586. doi: 10.1371/journal.pone.0022586 (PMC3142187; doi:10.1371/journal.pone.0022586)

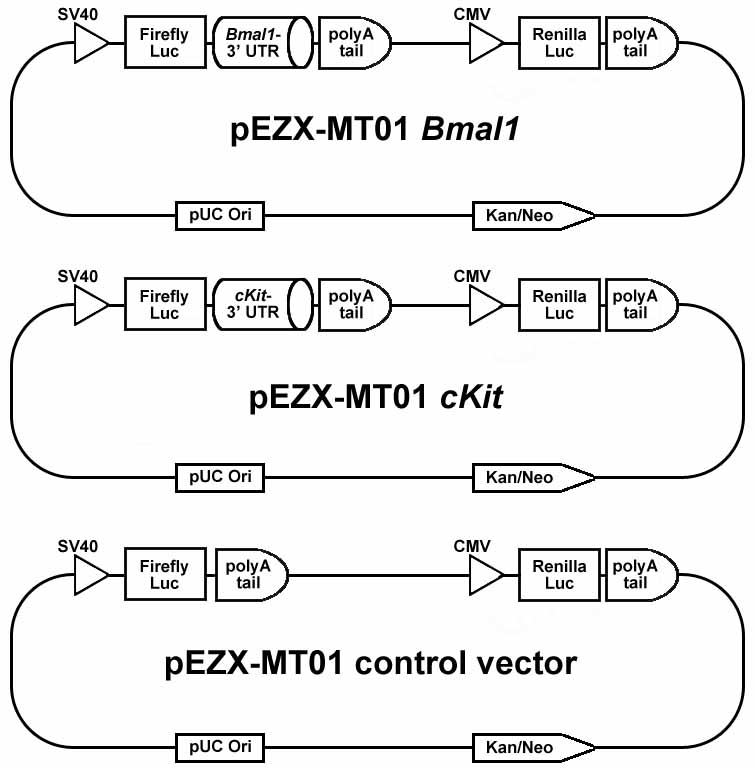

Supplement: Figure S2 — Design of pEZX-MT01 Bmal1 and cKit 3′ UTR luciferase reporter vectors. pEZX-MT01 dual luciferase reporter plasmid construct encodes a chimeric transcript containing the mouse Bmal1 or cKit 3′ UTR tagged to firefly luciferase coding sequence under control of an SV40 enhancer. In comparison, the miRNA 3′ UTR target control vector lacks a 3′ UTR target downstream of the pEZX-MT01 firefly luciferase coding sequence. All vectors contain a kanamycin resistance cassette for selection of bacterial transformants stably expressing the pEZX-MT01 plasmid and the Renilla luciferase coding sequence transcribed under control of CMV promoter to normalize firefly luciferase signal intensities across samples. (TIF) [file pone.0022586.s002.tif]

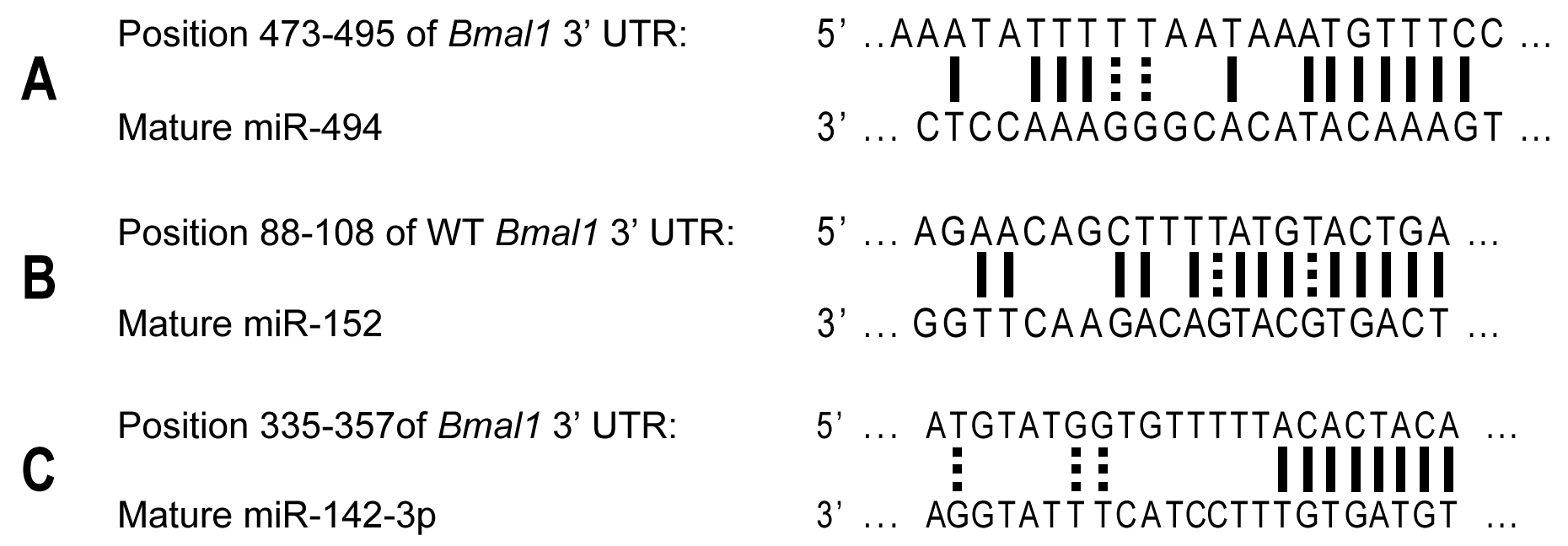

Supplement: Figure S3 — Predicted interactions between miR-494, miR-152 and miR-142-3p and the Bmal1 3′ UTR. Diagrammatic representation of predicted interactions between mature (A) miR-494, (B) miR-152 and (C) miR-142-3p with complementary regions of the wild-type Bmal1 3′ UTR. Dotted lines indicate potential stabilizing interactions between guanine and thymine bases. (TIF) [file pone.0022586.s003.tif]
